# Supplementary material for: Capital market liberalization and corporate environmental performance: Evidence from the Shanghai–Hong Kong Stock Connect
Source: PLoS One. 2024 Nov 8;19(11):e0312461. doi: 10.1371/journal.pone.0312461 (PMC11548747; doi:10.1371/journal.pone.0312461)
Supplement: Attachment — Submitted filename: Response to Reviewers.docx [file pone.0312461.s001.docx]

# **Re: Response for manuscript”****Capital market liberalization and corporate environmental performance - Evidence from the Shanghai-Hong Kong Stock Connect”**

Dear Reviewers,

Thank you very much for your time involved in reviewing the manuscript and your very encouraging comments on the merits.

We also appreciate your clear and detailed feedback and hope that the explanation has fully addressed all of your concerns. In the remainder of this letter, we discuss each of your comments individually along with our corresponding responses.

To facilitate this discussion, we first retype your comments in *italic font* and then present our responses to the comments.

**Comment 1:**

In the current version of the manuscript, the literature review could be improved by incorporating some relevant publications that have not been mentioned. Specifically, I recommend discussing the following papers in one or two paragraphs, as they can provide valuable insights and context for your study. For example, Bao and Huang (2021) provide evidence on the role of FinTech in the context of a crisis, which could be relevant to understanding the impact of capital market liberalization on corporate environmental performance. Chen et al. (2022) investigate the relationship between interlocking directorates and firm performance in China, which could provide insights into the governance mechanisms that may influence environmental performance. Chen et al. (2022) and Huang et al. (2022) both explore the dynamics of market connectivity and credit risk, respectively, which could be related to the mechanisms through which capital market liberalization affects environmental performance.

**Response 1:**

Thanks for your great suggestion on improving the accessibility of our manuscript. We have added the relevant literature.The details are as follows:

In relevant studies on corporate environmental performance,some studies are conducted at the macro level,Such as political connections[14],environmental protection tax[15],Climate risk[16],Low-carbon city pilot policy[17],and environmental regulation[18].Other studies focus on micro-level factors,Such as powerful CEOs[19], Multiple large shareholders[20];digital investment[21],corporate social responsibility[22],and genetic diversity on corporate boards[23].***Chen et al.(2022) find that the higher the degree and betweeness centrality of a firm in the interlocking directorate network, the stronger effects it have on firm performance[24].Better performance allows firms to have enough resources to reduce environmental pollution.Financial constraints are also an important factor affecting enterprises environmental performance.With the rapid development of FinTech in China, enterprises can obtain funds through a variety of ways, effectively alleviating their financing constraints.Bao and Huang（2021）determined that FinTech companies lent three times as much as banks during the epidemic, which also effectively alleviated the financing constraints of a large number of firms that were unable to obtain loans,which in turn improves corporate environmental performance[25].***

**Comment 2:**

*Please provide more details on the methodology used to select the 3248 samples of heavily-polluting enterprises. Explain the criteria used to define "heavily-polluting" and how these firms were identified.*

**Response 2:**

We have added the definition of the scope of heavy polluting enterprises. The details are as follows:***Heavily polluting enterprises referred to in this paper are enterprises in the thermal power, iron and steel, cement, electrolytic aluminum, coal, metallurgy, chemical, petrochemical, building materials, papermaking, brewing, pharmaceutical, fermentation, textile, tannery, and mining industries as defined in the "List of Listed Companies in Environmental Verification of Industry Classification and Management.***

**Comment 3:**

The manuscript would benefit from a more in-depth discussion of the mechanisms through which capital market liberalization affects environmental performance. In particular, please elaborate on the role of environmental assets, stock price volatility, and information disclosure quality in this relationship.

**Response 3:**

In Hypothesis development,we have added theoretical analysis.The details are as follows:

We expect that capital market liberalization affects corporate environmental performance by increasing environmental assets, reducing stock price volatility, and improving the quality of disclosure.First,capital market liberalization affects corporate environmental performance through investing environmental assets.***Investments in environmental assets are characterized by large investments, long lead times and low short-term benefits. As the social effect brought about by environmental asset investment is greater than the economic benefit, this also leads to the fact that in enterprises with high financing constraints, environmental asset investment will reduce the funds for other project investments, causing enterprise costs to rise and profits to decrease*.**Opening a capital market effectively integrates China's originally closed market with the global market,enhancing the efficiency of China's capital market[26,27].Good environmental performance can attract new investors ,and obtain sufficient funds to alleviate financing constraints.Feng et al. (2022) find that capital market liberalization reduces firms' financing constraints, thereby enhancing corporate green innovation.[28].Heavy polluting enterprises have enough money to invest in environmental assets to achieve lower pollutant emissions and environmental taxes, which in turn improves corporate environmental performance.

Second, capital market liberalization leads to higher synchronization of China's capital market with the overseas capital market, which to a certain extent bears the impact of large-scale capital flows and thus affects capital market stability [29]. In particular, after international risk events, which include the U.S. subprime mortgage crisis and the 2015 stock market crash in China ,substantial capital outflows from the capital market cause dramatic fluctuations in the market, increasing companies short-term financial risks.***Chen et al.(2022) find that stock prices have seen greater volatility in the U.S. subprime mortgage crisis as well as the 2015 subprime mortgage crisis[30].Based on the above background, firms need to have differentiated resources to dampen the impact of risk events on their share prices.*** *Environmental performance, as an important strategic resource, can alleviate the external market’s impact on firms to some extent[31].* ***In other words,enterprises with good environmental performance can relieve stock price fluctuations [31,32] to facilitate better access to funds for business growth through the capital markets.****Thus, firms should have the motivation and capability to enhance environmental performance.*

Finally, Capital market liberalization may facilitate the enhancement of corporate environmental performance by improving the information environment.Kim and Zhang(2014) find that when firms take on more social responsibility, they can curb the spread of negative news by increasing the transparency of financial reporting[33].Nie et al.(2023) find that Capital market liberalization enhances corporate ESG disclosure and satisfies the needs of investors[34]. Thus,the pressure to disclose environmental information motivates heavily polluting firms to enhance their environmental protection efforts, thereby improving corporate environmental performance.

Based on the above analysis, we propose the following hypothesis:

**Hypothesis1: Capital market liberalization improves corporate environmental performance**

In Mediation analysis,We provide an in-depth analysis of the mechanisms by which capital market freedom affects environmental performance.The details are as follows:

4.5.1 Environmental assets（EA）

The results of the mediating effects of environmental assets are presented in Table 8.As can be seen,the estimated coefficient of List*Post in column (2) is significantly positive,which indicates that capital market liberalization prompts heavily polluting firms to increase investment in environmental assets. When List*Post and EA are regressed together as environmental performance explanatory variables, the regression coefficient of List*Post decreases to 0.035 compared to column (1), while the regression coefficient of EA remains significant.Based on this calculations,the mediating effect of EA is 46.7%.The above analysis suggests that the mediating effect of environmental assets holds. *It indicates that capital market liberalization pushes heavily polluting enterprises to increase their investment in environmental assets, which in turn leads to an improvement in corporate environmental performance.*

4.5.2 Stock price volatility(VOL)

The results of the mediating effects of stock price volatility are presented in Table 9.As can be seen,the estimated coefficient of List*Post in column (2) is significantly negative,which indicates that capital market liberalization significantly reduces stock price volatility.When List*Post and VOL are regressed together as environmental performance explanatory variables, the regression coefficient of List*Post decreases to 0.052 compared to column (1), while the regression coefficient of VOL remains significant.Based on this calculations,the mediating effect of VOL is 13.3%.This suggests that VOL mediates the effect of capital market liberalization on environmental performance.*It indicates that capital market liberalization exacerbates the impact of risky events on stock prices, while good environmental performance can effectively alleviate this impact.This also provides firms with access to stable funding from the capital market, which contributes to their environmental performance.*

4.5.3 Information disclosure quality（DQ）

We also report results for the mediating effect of disclosure quality in Table 10.The regression coefficient of List*Post is significantly positive in columns (2) ,which indicates that capital market liberalization prompts heavily polluting firms to improve information disclosure quality.When List*Post and IDQ are regressed together as environmental performance explanatory variables, the coefficient of List*Post decreases to 0.023 compared to column (1), while the regression coefficient of DQ remains significant.Based on this calculations,the mediating effect of DQ is 61.7%.This suggests that DQ mediates the effect of capital market liberalization on environmental performance.*It indicates that capital market liberalization enhances the quality of information disclosure by heavy polluters, reduces the degree of information asymmetry between corporate management and investors, and alleviates the financing constraints due to information asymmetry thereby promoting environmental performance.*

**Comment 4:**

Consider conducting additional robustness checks to strengthen the validity of your findings. For example, you could perform a placebo test or use alternative measures of environmental performance.

**Response 4:**

We have added a placebo test.The details are as follows:

***In order to verify that the improvement in environmental performance of heavy polluting enterprises is due to the implementation of SH-HKSC and not due to other factors.We advance the implementation of the SH-HKSC policy by two years, and if the coefficient of List*Post is significant, indicating that the improvement of environmental performance of heavy polluters is not related to the opening of the capital market.The estimated coefficients of List*Post shown in Columns (1) and (2) of Table 7 are insignificant, indicating that indicating that the improvement in environmental performance of heavy polluting enterprises is due to capital market liberalization, thus validating the conclusions of this study.***

***Table 7:Placebo test***

| ***Variable*** | ***EID(1)*** | ***HEID(2)*** |
| --- | --- | --- |
| ***List*Post*** | ***-0.039（-0.36）*** | ***0.015（1.30）*** |
| ***Constant*** | ***1.020***（6.21）*** | ***0.524***（11.25）*** |
| ***Controls*** | ***YES*** | ***YES*** |
| ***Year/Firm Fixed effect*** | ***YES*** | ***YES*** |
| ***R^2^*** | ***0.206*** | ***0.247*** |
| ***N*** | ***3248*** | ***3248*** |

***T-statistics for the regression coefficients are in parentheses*, **, and *** represent statistical significance at the 10%, 5%, and 1% levels,respectively.***

We would like to take this opportunity to thank you for all your time involved and this great opportunity for us to improve the manuscript. We hope you will find this revised version satisfactory.

# **Funding Statement:**This study was supported by Philosophy and Social Science Project of Colleges and Universities in Jiangsu Province (2023SJYB2339). Suqian College Youth Research Fund Project (2023XQNA10).

Sincerely,

Zhong
