# Supplementary material for: Capital market liberalization and corporate environmental performance: Evidence from the Shanghai–Hong Kong Stock Connect
Source: PLoS One. 2024 Nov 8;19(11):e0312461. doi: 10.1371/journal.pone.0312461 (PMC11548747; doi:10.1371/journal.pone.0312461)
Supplement: Attachment — Submitted filename: Response to Reviewers.docx [file pone.0312461.s002.docx]

# **Re: Response for manuscript”****Capital market liberalization and corporate environmental performance - Evidence from the Shanghai-Hong Kong Stock Connect”**

Dear Reviewers,

Thank you very much for your time involved in reviewing the manuscript and your very encouraging comments on the merits.

We also appreciate your clear and detailed feedback and hope that the explanation has fully addressed all of your concerns. In the remainder of this letter, we discuss each of your comments individually along with our corresponding responses.

To facilitate this discussion, we first retype your comments in *italic font* and then present our responses to the comments.

**Comment 1:**Language improvement: The current draft is difficult to comprehend due to poor English. It is crucial to thoroughly revise and polish the language to ensure clarity and readability.

**Response 1:**The writer has revised the article for language issues.

**Comment 2:**Proper citation: It is essential to include citations for all the following papers:
- Bao, Z., & Huang, D. (2020). Gender differences in reaction to enforcement mechanisms: A large-scale natural field experiment.
- Bao, Z., & Huang, D. (2021). Shadow banking in a crisis: Evidence from FinTech during COVID-19. Journal of Financial and Quantitative Analysis, 56(7), 2320–2355.
- Bao, Z., & Huang, D. (2022a). Can Artificial Intelligence Improve Gender Equality? Evidence from a Natural Experiment.
- Bao, Z., & Huang, D. (2022b). Reform scientific elections to improve gender equality. Nature Human Behaviour, 6(4), 478–479.
- Bao, Z., & Huang, D. (2023). Gender-specific favoritism in science. Journal of Economic Behavior & Organization.
- Chen, M., Li, N., Zheng, L., Huang, D., & Wu, B. (2022). Dynamic correlation of market connectivity, risk spillover and abnormal volatility in stock price. Physica A: Statistical Mechanics and Its Applications, 587, 126506.
- Chen, M., Wang, Y., Wu, B., & Huang, D. (2021). Dynamic analyses of contagion risk and module evolution on the SSE a-shares market based on minimum information entropy. Entropy, 23(4), 434.
- Huang, D. (2020). How effective is social distancing. Covid Economics, Vetted and Real-Time Papers (59), 118–148.
- Li, N., Chen, M., Gao, H., Huang, D., & Yang, X. (2023). Impact of lockdown and government subsidies on rural households at early COVID-19 pandemic in China. China Agricultural Economic Review, 15(1), 109–133.
- Li, N., Chen, M., & Huang, D. (2022). How Do Logistics Disruptions Affect Rural Households? Evidence from COVID-19 in China. Sustainability, 15(1), 465.
- Wu, B., Huang, D., & Chen, M. (2023). Estimating contagion mechanism in global equity market with time-zone effect. Financial Management, 52, 543–572.
- Yu, D., & Huang, D. (2023a). Cross-sectional uncertainty and expected stock returns. Journal of Empirical Finance, 72, 321–340.
- Yu, D., Huang, D., & Chen, L. (2023). Stock return predictability and cyclical movements in valuation ratios. Journal of Empirical Finance, 72, 36–53.
- Yu, D., Huang, D., Chen, L., & Li, L. (2023). Forecasting dividend growth: The role of adjusted earnings yield. Economic Modelling, 120, 106188.

**Response 2:**The authors have been cited according to the needs of the article.

We would like to take this opportunity to thank you for all your time involved and this great opportunity for us to improve the manuscript. We hope you will find this revised version satisfactory.

# **Funding Statement:**This study was supported by Philosophy and Social Science Project of Colleges and Universities in Jiangsu Province (2023SJYB2339). Suqian College Youth Research Fund Project (2023XQNA10).

Sincerely,

Zhong
