# Supplementary material for: Capital market liberalization and corporate environmental performance: Evidence from the Shanghai–Hong Kong Stock Connect
Source: PLoS One. 2024 Nov 8;19(11):e0312461. doi: 10.1371/journal.pone.0312461 (PMC11548747; doi:10.1371/journal.pone.0312461)
Supplement: Attachment — Submitted filename: Response to Reviewers.docx [file pone.0312461.s003.docx]

# **Re: Response for manuscript”****Capital market liberalization and corporate environmental performance - Evidence from the Shanghai-Hong Kong Stock Connect”**

Dear Reviewers,

Thank you very much for your time involved in reviewing the manuscript and your very encouraging comments on the merits.

We also appreciate your clear and detailed feedback and hope that the explanation has fully addressed all of your concerns. In the remainder of this letter, we discuss each of your comments individually along with our corresponding responses.

**Comment 1:**

The contribution of this study is not clear, and the introduction section is relatively brief. Sha et al. (2022), Zhang et al. (2023), Deng et al. (2023), Sha et al., 2022, and He et al. (2024) have thoroughly demonstrated the relationship between capital market opening and environmental performance, and have concluded that capital market opening improve environmental performance. Therefore, this greatly undermines the theoretical contribution of this study. This makes it difficult for the first innovation point of this study to continue, in other words, this work does not substantively contribute to the existing literature. Although this study attempts to differentiate from existing literature through mechanism research (environmental assets, stock price volatility, and information disclosure quality), these mechanisms can essentially be explained by the mitigation of information asymmetry (Zhang et al., 2022; Liu & Niu, 2023; Yi, 2023), investor environmental concern (Sha et al., 2022; Deng et al., 2023) and information disclosure (Qing, 2022) proposed in existing literature, weakening the contribution of this study. The authors need to elaborate on the differences with existing literature in detail and briefly outline the introduction section, which includes motivation, theoretical support, brief literature/literature gap, research objectives, research contributions, brief findings within a paragraph, and research structure.
**Response 1:**

Thanks for your great suggestion on improving the accessibility of our manuscript. We have added the relevant literature.The details are as follows:

Previous studies have also examined the relationship between market liberalization and environmental performance[5-6]. However, their selection of samples and the quantification of environmental performance lacked specificity. This research is based on heavily polluting enterprises as the research samples, so the sample selection is more targeted. Besides，we use emission charges and expenditures on environmental taxes to quantify corporate environmental performance, so the research results will be closely related to its business-level environmental performance.

**Comment 2:**

The research motivation of this study is insufficient. Based on the realistic background, the authors need to identify the problems existing in environmental performance in China in the introduction section and explain why the Shanghai-Hong Kong Stock Connect can alleviate these problems to a certain extent, that is, to elucidate the causal logic between the Shanghai-Hong Kong Stock Connect and corporate environmental performance. Considering the literature background, the research topic, research methods, and research conclusions of this study do not fill the gaps in existing literature. Therefore, the authors need to delve deeper into the relationship between capital market opening and environmental performance and identify more specific issues or problems that have been overlooked by the academic community.
**Response 2:**

Investors use corporate environmental performance as a crucial indicator to understand companies’ sustainable development[3],evaluate investment value[4], and reflect the corporate value in the stock price. Before liberalization, Chinese government has developed many legal systems for environmental protection, but environmental problems remain serious. ***The reasons for the above problems are mainly manifested in the following aspects.On the one hand, some enterprises lack sufficient motivation to reduce the emission of pollutants facing pressure from the market, technology and capital. On the other hand some enterprises do not have enough knowledge about the environmental pollution problems, which leads to the ineffective solution. The liberalization of capital market provides an opportunity to solve the problem of environmental pollution. Some studies have demonstrated that capital market liberalization can improve corporate environmental performance[5-6], but there is a lack of specificity in sample selection and the conclusions may lack applicability. This study examines the relationship between capital market liberalization and corporate environmental performance using heavily polluting firms as research samples. Results show that capital market liberalization significantly enhances corporate environmental performance. The mechanism of action test indicates that capital market liberalization enhances corporate environmental performance by enhancing investment in environmental assets, reducing stock volatility, and improving the quality of information disclosure. The heterogeneity test finds that capital market liberalization has significant effect on corporate environmental performance in Non-SOEs and firms with low internal control.***

**Comment 3:**

In terms of hypothesis development, this study lacks a comprehensive literature review and theoretical foundation and does not elaborate on the relationship between capital market opening and the mechanism variables. Therefore, we recommend that the authors cite relevant literature on the economic consequences of capital market opening (Chen et al., 2023; Wang et al., 2024) and literature on factors influencing green innovation (Takalo et al., 2021; Tseng et al., 2013; Xiang et al., 2022) to incorporate research conclusions into the logical framework of this study.
**Response 3:**

In Literature Review,we have added an analysis of the relevant literature.The details are as follows:

2.1.1 Capital market liberalization

Existing literature on the economic consequences of capital market liberalization focuses mainly on the operation of capital markets,the behavior of enterprises, and the development of the real economy.First,capital market liberalization has introduced mature value investors to developing countries[6], which can guide investors to make value investments[7], improve the information content of stock prices[8], reduce the heterogeneous volatility of stock markets[9], and thus improve stock market stability and market effectiveness[10].Second, market transactions conducted by foreign investors can affect corporate decision-making behavior, such as improving the efficiency of corporate investment[6], reducing investment in inefficient labor[9],*reducing commercial bank risk[11], reducing firms’earnings management[12],*and promoting corporate social responsibility[13].Finally, foreign investors can optimize corporate governance mechanisms to curb aggressive financial asset allocation by enterprises and enhance the quality of financial services to the real economy[14].

2.1.2 Corporate environmental performance

*Firstly, the factors influencing the corporate environmental performance.*In relevant studies on corporate environmental performance,some studies are conducted at the macro level,Such as political connections[15],environmental protection tax[16],Climate risk[17],Low-carbon city pilot policy[18],and environmental regulation[19].Other studies focus on micro-level factors,Such as powerful CEOs[20], Multiple large shareholders[21];digital investment[22],corporate social responsibility[23],and genetic diversity on corporate boards[24].*Second, the importance of corporate environmental performance.Some scholars argue that green innovation (GI) can be used as an alternative to corporate environmental performance.Takalo et al.(2021) suggest that A large number of organizations and communities have been directed towards green innovation as a strategy to achieve environmental protection and economic growth[25].Third, evaluating enterprises environmental performance .Tseng et al. (2013) assessed the green innovation practices of printed circuit board manufacturing companies in Taiwan in terms of four dimensions: management, process, product, and technological innovation,and also found that management innovation is a key driver of green innovation[26].*

**Comment 4:**

There are several issues in the empirical test of this study: (1) The time of enterprises' entry into the Shanghai-Hong Kong Stock Connect and the Shenzhen-Hong Kong Stock Connect differs. Therefore, the paper presents a staggered Difference-in-Differences (DID) research question, and this paper's approach of using 2014 as the base year is unsound. (2) The robustness tests of the paper are relatively weak, lacking thorough robustness tests. (3) Furthermore, the paper investigates the relationship between capital market liberalization and environmental performance by selecting heavily polluting enterprises among Chinese listed companies, which introduces sample selection bias. We suggest the author adopts the Heckman two-stage method to address endogeneity and employ alternative explanations to avoid the impact of concurrent policy shocks.

**Response 4:**

*First , this study selects the companies of Shanghai-Hong Kong Stock Connect as the research sample, and does not select the companies of Shenzhen-Hong Kong Stock Connect as the research sample. The opening of Shanghai-Hong Kong Stock Connect was in 2014.*

*Second，We added the Heckman test.*

*4.3.6 Heckman test*

*We use the Heckman two-stage model to control for the problem of self-selection of samples. Referring to Zhang (2023),In addition to the original control variables, we add variables that may affect the selection of firms into the SH-HKSC , including firm value (TobinQ), Listage(Age), stock turnover(Turnover), and dividend payout ratio (Dividen). The first stage probit regression results are shown in column 1 of Table 8. The results of Heckman's second stage regression are in Column 2 and 3 of Table 8, and the inverse Mills coefficient (imr) is significant at the 1 per cent level.The regression coefficients for List*Post remain significant when we control for relevant control variables, time effects, and firm effects, consistent with the baseline regression results.*

**Table 8. Heckman test**

| *Variable* | *SH-HKSC(1)* | *EID(2)* | *HEID(3)* |
| --- | --- | --- | --- |
| *List*Post* |  | *0.060***(11.05)* | *-0.059***(-6.94)* |
| *Size* | *0.025*(1.20)* | *0.023**(3.06)* | *0.019*(2.64)* |
| *Sg* | *0.126**(4.28)* | *0.102*(2.65)* | *0.096*(4.36)* |
| *Intan* | *0.001(0.10)* | *0.001(0.00)* | *0.002(0.00)* |
| *LEV* | *-0.032*(1.34)* | *-0.114**(6.95)* | *-0.157**(11.03)* |
| *DL* | *0.241***(11.52)* | *0.138***(16.30)* | *0.206***(15.30)* |
| *FS* | *-0.057**(-5.03)* | *-0.048*(-2.92)* | *-0.062*(-3.61)* |
| *INS* | *0.359***(22.03)* | *0.267***(19.63)* | *0.168***(16.30)* |
| *TobinQ* | *0.143***(9.06)* |  |  |
| *Age* | *0.010*(3.03)* |  |  |
| *Turnover* | *0.031(1.37)* |  |  |
| *Dividen* | *0.548***(33.64)* |  |  |
| *imr* |  | *1.305***(36.02)* | *0.962***(30.87)* |
| *Year/Firm Fixed effect* | *NO* | *YES* | *YES* |
| *R^2^* | *0.103* | *0.268* | *0.251* |
| *N* | *3248* | *3248* | *3248* |

**Comment 5:**

In the mechanism testing section, the authors need to present the measurement methods for each mechanism variable and propose relevant hypotheses for the mechanisms in the literature review and hypotheses.

**Response 5:**

First, we quantified each mediating variable.second，we propose relevant hypotheses for the mechanisms in the hypotheses.

| **Variable Name** | **Variable Symbol** | **Variable Definition** |
| --- | --- | --- |
| Corporate environmental performance | EID | the ratio of the logarithm of emissions charges to the logarithm of operating revenues |
|  | HEID | the ratio of the logarithm of corporate environmental taxes to the logarithm of operating revenues |
| Underlying Stocks of Shanghai-Hong Kong Stock Connect | List | List is a company dummy variable,which is 1 if the firm belong to the SH-HKSC,and 0 otherwise |
| Shanghai-Hong Kong Stock Connect Launch Time | Post | Post is a time dummy variable, which is 1 when year is after 2014，and 0 otherwise. |
| ***Environmental assets*** | ***EA*** | ***the ratio of environmental assets to total assets*** |
| ***Stock price volatility*** | ***VOL*** | ***the standard deviation of daily stock returns over the quarter*** |
| ***Information disclosure quality*** | ***DQ*** | ***DQ comes from CSMAR.The indicator is categorized into four levels and assigned values from 1 ( bad ) to 4 ( good ).*** |

First,capital market liberalization affects corporate environmental performance through investing environmental assets.Investments in environmental assets are characterized by large investments, long lead times and low short-term benefits. As the social effect brought about by environmental asset investment is greater than the economic benefit, this also leads to the fact that in enterprises with high financing constraints, environmental asset investment will reduce the funds for other project investments, causing enterprise costs to rise and profits to decrease.Opening a capital market effectively integrates China's originally closed market with the global market,enhancing the efficiency of China's capital market[24,25].Good environmental performance can attract new investors ,and obtain sufficient funds to alleviate financing constraints.Feng et al. (2022) find that capital market liberalization reduces firms' financing constraints, thereby enhancing corporate green innovation.[26].Heavy polluting enterprises have enough money to invest in environmental assets to achieve lower pollutant emissions and environmental taxes, which in turn improves corporate environmental performance.

Based on the above analysis, we propose the following hypothesis:

***Hypothesis 1: Capital market liberalization enhances environmental performance by increasing investment in environmental assets.***

Second, capital market liberalization leads to higher synchronization of China's capital market with the overseas capital market, which to a certain extent bears the impact of large-scale capital flows and thus affects capital market stability [27]. In particular, after international risk events, which include the U.S. subprime mortgage crisis and the 2015 stock market crash in China ,substantial capital outflows from the capital market cause dramatic fluctuations in the market, increasing companies short-term financial risks.Environmental performance, as an important strategic resource, can alleviate the external market’s impact on firms to some extent[28]. In other words,enterprises with good environmental performance can relieve stock price fluctuations to facilitate better access to funds for business growth through the capital markets[29,30,31].Thus, firms should have the motivation and capability to enhance environmental performance.

***Hypothesis 2: Capital market liberalization enhances environmental performance by reducing stock price volatility***

Finally, Capital market liberalization may facilitate the enhancement of corporate environmental performance by improving the information environment.Kim and Zhang(2014) find that when firms take on more social responsibility, they can curb the spread of negative news by increasing the transparency of financial reporting[32].Nie et al.(2023) find that Capital market liberalization enhances corporate ESG disclosure and satisfies the needs of investors[33]. Thus,the pressure to disclose environmental information motivates heavily polluting firms to enhance their environmental protection efforts, thereby improving corporate environmental performance.

Based on the above analysis, we propose the following hypothesis:

***Hypothesis 3: Capital market liberalization improves corporate environmental performance by improving the quality of disclosure.***

**Comment 6:**

This study lacks explanations for the regression coefficients and economic significance of the results. In the heterogeneity analysis, the authors need to present in the main text why the regression results show heterogeneity in different groups, that is, the inherent connection between the grouping variable and the baseline regression. Additionally, the study did not test the differences in coefficients between the two groups, which makes it difficult to rigorously prove the heterogeneity of the results between the two groups.
**Response 6:**
4.4. Heterogeneity analysis

Table 8 shows the regression results of the firm heterogeneity.We categorize firms into state-owned and non-state-owned firms(SOEs and Non-SOEs), and firms with high internal controls and low internal controls. The List*Post coefficients are significantly higher for Non-SOEs and firms with low internal control than for SOEs and firms with high internal control, respectively.*Firstly, compared to SOEs, non-SOEs have more incentives to illustrate the effectiveness of their environmental protection through good environmental performance in order to gain government policy support and investors' attention.Secondly, firms with low internal control are more willing to obtain investment from overseas investors through good environmental performance under the pressure of capital market liberalisation compared to firms with high internal control.*

**Comment 7:**

The conclusion section of this study is relatively brief and needs to provide policy recommendations based on the regression results.

**Response 6:**

This study provides new evidence on the economic effects of capital market liberalization and expands the research related to corporate environmental performance.*For Governments,Drawing on the experience of the smooth implementation of the SH-HKSC, the government should continue to promote the opening of the capital market in a prudent and reasonable manner, gradually expand and deepen the level of opening of the capital market, accelerate the docking of the capital market with the international capital market[5], draw on the capital market systems of international developed countries and regions, and actively draw on their experience in order to establish and improve the capital market system, and actively make use of the international capital, talents and systems to provide a good and effective capital market environment for the green development of enterprises and even for the green transformation of the economy and society.For enterprises, it is important to take advantage of the opportunities arising from the opening of the capital market to raise awareness of corporate social responsibility and to achieve sustainable development by improving environmental performance.*

We would like to take this opportunity to thank you for all your time involved and this great opportunity for us to improve the manuscript. We hope you will find this revised version satisfactory.

Sincerely,

Zhong
