# Supplementary material for: Capital market liberalization and corporate environmental performance: Evidence from the Shanghai–Hong Kong Stock Connect
Source: PLoS One. 2024 Nov 8;19(11):e0312461. doi: 10.1371/journal.pone.0312461 (PMC11548747; doi:10.1371/journal.pone.0312461)
Supplement: Attachment — Submitted filename: Response to Reviewers.docx [file pone.0312461.s004.docx]

# **Re: Response for manuscript”****Capital market liberalization and corporate environmental performance - Evidence from the Shanghai-Hong Kong Stock Connect”**

Dear Reviewers,

Thank you very much for your time involved in reviewing the manuscript and your very encouraging comments on the merits.

We also appreciate your clear and detailed feedback and hope that the explanation has fully addressed all of your concerns. In the remainder of this letter, we discuss each of your comments individually along with our corresponding responses.

**Comment 1:**

Please answer the reviewers comments and highlight the changes made in yellow or different font color to ease the review process and improve the manuscript, as it has area to improve in all section, especially introduction, and hypothesis development. Moreover, there is an recent literature with respect to the environment studies variables as discussed in following studies. These studies will help you to improve your arguments and methodology.
**Response 1:**

Thanks for your great suggestion on improving the accessibility of our manuscript. We have added the relevant literature.The details are as follows:

Investors use corporate environmental performance as a crucial indicator to understand companies’ sustainable development[3],evaluate investment value[4], and reflect the corporate value in the stock price. Before liberalization, Chinese government has developed many legal systems for environmental protection, but environmental problems remain serious. The reasons for the above problems are mainly manifested in the following aspects.On the one hand, some enterprises lack sufficient motivation to reduce the emission of pollutants facing pressure from the market, technology and capital. On the other hand some enterprises do not have enough knowledge about the environmental pollution problems, which leads to the ineffective solution. The liberalization of capital market provides an opportunity to solve the problem of environmental pollution.Some studies have demonstrated that capital market liberalization can promote corporate environmental performance[5-6], and there is also to be a study that shows that capital market liberalization capital market liberalization promotes green innovation in China and has a stronger impact on green invention patents than on green utility model patents[7],but there is a lack of specificity in sample selection and the conclusions may lack applicability. This study examines the relationship between capital market liberalization and corporate environmental performance using heavily polluting firms as research samples. Results show that capital market liberalization significantly enhances corporate environmental performance. The mechanism of action test indicates that capital market liberalization enhances corporate environmental performance by enhancing investment in environmental assets, reducing stock volatility, and improving the quality of information disclosure. The heterogeneity test finds that capital market liberalization has significant effect on corporate environmental performance in Non-SOEs and firms with low internal control.

2.1.2 Corporate environmental performance

Firstly, the factors influencing the corporate environmental performance.In relevant studies on corporate environmental performance,some studies are conducted at the macro level,Such as political connections[16],environmental protection tax[17],Climate risk[18],Low-carbon city pilot policy[19],and environmental regulation[20].Other studies focus on micro-level factors,Such as powerful CEOs[21], Multiple large shareholders[22];digital investment[23],corporate social responsibility[24], managers capabilities[25],board gender diversity[26],and genetic diversity on corporate boards[27].Zahid et al.(2024) suggested that managers with strong management skills exhibit a positive correlation with their company's ESG performance and its subcomponents[25].Zahid et al.(2023) found that more women who served on corporate boards enhanced the company's environmental performance and disclosures while limiting greenwashing behavior. Second, the importance of corporate environmental performance.Some scholars argue that green innovation (GI) can be used as an alternative to corporate environmental performance.Takalo et al.(2021) suggested that A large number of organizations and communities have been directed towards green innovation as a strategy to achieve environmental protection and economic growth[28].Third, evaluating enterprises environmental performance .Tseng et al. (2013) assessed the green innovation practices of printed circuit board manufacturing companies in Taiwan in terms of four dimensions: management, process, product, and technological innovation,and also found that management innovation is a key driver of green innovation[29].

We would like to take this opportunity to thank you for all your time involved and this great opportunity for us to improve the manuscript. We hope you will find this revised version satisfactory.

Sincerely,

Zhong
